# Safety and efficacy of day anterior cervical discectomy and fusion procedure for degenerative cervical spondylosis: a retrospective analysis

**DOI:** 10.1186/s12891-024-07356-7

**Published:** 2024-03-19

**Authors:** Long Tang, Yu Chen, Fandong Wang, Yuanbin Liu, Zhaojun Song, Miao Wang, Yong Zhou, Huiyi Liu, Jiazhuang Zheng

**Affiliations:** 1 Department of Spine Surgery, Suining Central Hospital, 127 Desheng West Road, Suining, Sichuan Province 629000 China; 2 Department of Neurosurgery, Klinikum Rechts Der Isar, Technical University of Munich, Ismaninger Straße 22, Munich, 81675 Germany

**Keywords:** Cervical spondylosis, Diskectomy, Spinal fusion, Day surgery, Clinical efficacy

## Abstract

**Objective:**

Our study aimed to develop a day anterior cervical discectomy and fusion (ACDF) procedure to treat degenerative cervical spondylosis (DCS). The goal was to analyze its clinical implications, safety, and early effects to provide a better surgical option for eligible DCS patients.

**Methods:**

A retrospective analysis was performed to identify DCS patients who underwent day ACDF from September 2022 to August 2023. The operative time, intraoperative blood loss, postoperative drainage, preoperative and postoperative visual analog scale (VAS) scores, neck disability index (NDI) scores, Japanese Orthopedic Association (JOA) scores, JOA recovery rate (RR), incidence of dysphagia-related symptoms, 30-day hospital readmission rate, and incidence of other complications were recorded to evaluate early clinical outcomes. Radiography was performed to assess the location of the implants, neurological decompression, and cervical physiological curvature.

**Results:**

All 33 patients (23 women and 10 men) underwent successful surgery and experienced significant symptomatic and neurological improvements. Among them, 26 patients underwent one-segment ACDF, 5 underwent two-segment ACDF, and 2 underwent three-segment ACDF. The average operative time was 71.1 ± 20.2 min, intraoperative blood loss was 19.1 ± 6.2 mL, and postoperative drainage was 9.6 ± 5.8 mL. The preoperative VAS and NDI scores improved postoperatively (7.1 ± 1.2 vs. 3.1 ± 1.3 and 66.7% ± 4.8% vs. 24.1% ± 2.5%, respectively), with a significant difference (*P* < 0.01). Moreover, the preoperative JOA scores improved significantly postoperatively (7.7 ± 1.3 vs. 14.2 ± 1.4; *P* < 0.01) with an RR of 93.9% in good or excellent. Postoperative dysphagia-related symptoms occurred in one patient (3.0%). During the follow-up period, no patient was readmitted within 30 days after discharge; however, an incisional hematoma was reported in one patient on the 6th day after discharge, which was cured by pressure dressing. The postoperative radiographs revealed perfect implant positions and sufficient nerve decompression in all patients. Furthermore, the preoperative cervical physiological curvature improved significantly after the operation (14.5° ± 4.0° vs. 26.3° ± 5.4°; *P* < 0.01).

**Conclusions:**

Day ACDF has good safety and early clinical efficacy, and it could be an appropriate choice for eligible DCS patients.

## Introduction

Degenerative cervical spondylosis (DCS) is a chronic degenerative process of the cervical spine, mainly presenting as radicular neck or arm pain. It is the leading cause of myelopathy and radiculopathy in adults and is caused by degenerative disc disease or cervical spondylotic myelopathy (CSM) [1–3]. The prevalence of DCS is approximately 85% in patients aged ≥ 60 years [4]. When the clinical symptoms are severe and progressive or conservative therapy fails, surgery should be considered [5].

Smith and Cloward [6] initially reported the anterior cervical discectomy and interbody fusion (ACDF) procedure in the 1950s. This is the classic procedure for treating DCS, with approximately 132,000 operations performed in the United States annually [7], and this number is increasing steadily [8]. Generally, patients with ACDF stay in the hospital for 2–4 days postoperatively. The principal advantage of the postoperative stay is to closely monitor the patient’s neurologic status and any complications, such as dysphagia, hematoma, and respiratory insufficiency [9, 10]. The procedure is generally successful, and the incidence of complications is low, especially with the advancement of spinal instrumentation tools and techniques [11, 12] and the application of enhanced recovery after surgery [8, 13–15].

Over the past few decades, the technical level of spinal surgery has improved gradually; hence, spinal surgeries, including ACDF, are being increasingly performed as day procedures in some developed countries such as the European countries and the United States [16, 17]. Villavicencio et al. reported shorter total hospitalization time and quicker return to home with the day ACDF procedure [18]. However, in China, ACDF is mainly performed as an inpatient surgery, and only a few relevant studies have reported on either day or outpatient ACDF surgery. Therefore, our study aimed to develop a day ACDF procedure to treat DCS and to analyze its clinical implications, safety, and early effects to provide a better surgical option to eligible DCS patients.

## Materials and methods

### General information

In total, 33 patients (10 men and 23 women) with DCS had undergone one-, two-, or three-segment ACDF procedures at our academic Spine Surgery Department from September 2022 to August 2023. DCS was confirmed using cervical spine radiography, computed tomography (CT), and magnetic resonance imaging (MRI). Each patient underwent conservative treatment for at least 6 months with the following measurements: For patients with cervical spondylotic radiculopathy (CSR) and those with CSM without obvious spinal cord dysfunction, we mainly provided analgesia, muscle relaxation, anti-inflammatory with glucocorticoids, nutritional nerves, and physical therapy before surgery, but the latter should try to avoid massage or strenuous activities. Then, for CSM with obvious spinal cord dysfunction, surgery should be performed as soon as possible once diagnosed. However, none of them responded to conservative therapy. Patients with CSR mainly presented with pain in one upper limb, and a few with numbness. Patients with CSM mainly presented with neurological dysfunction such as unstable gait and limb weakness with physical examination showing that some patients had decreased muscle strength, but no muscle atrophy was found. Table 1 shows the inclusion criteria of this study. The mean age of the patients was 53.4 ± 9.1 years (36–67 years), and 42 Zero-Ps (Synthes GmbH) were implanted in the target segments. The general characteristics data are presented in Table 2. Informed consent was obtained from all patients.

**Table 1:** Day ACDF inclusion criteria

Candidates for Day ACDF Surgery Include Patients with All the Following Characteristics
1. Age ≤ 70 years old
2. Diagnosed as DCS with initial surgery
3. Surgical level located at C3/4–6/7
4.A single operation segment ≤ 3
5. The spinal canal invasion rate measured on MRI < 50%
6. Failed conservative treatment for 6 months or symptoms worsen
7. No obvious cardiopulmonary and respiratory system major underlying diseases and bleeding disorders
8. ASA score < III
9. BMI < 35 kg/m^2^
10. Agree to perform daytime ACDF

*ACDF* Anterior Cervical Discectomy and Fusion, *DCS* Degenerative Cervical Spondylosis, *MRI* Magnetic Resonance Imaging, *ASA* American Society of Anesthesiologists, *BMI* Body Mass Index

**Table 2:** Demographic data of the day ACDF cases (*n,* $\overline{x }$±S)

Characteristics	No
Gender (Male/Female)	10/23
Age (Years) History (Months)	53.4 ± 9.1 23.0 ± 10.6
Height(cm)	160.7 ± 6.7
Weight (Kg)	61.5 ± 7.2
BMI (kg/m^2^)	23.8 ± 2.7
ASA	II
VAS	7.1 ± 1.2
JOA	7.7 ± 1.3
NDI(%)	66.7 ± 4.8
Operation level:	
C4/5	11
C5/6	11
C6/7	4
C4/5, 5/6	3
C5/6, 6/7	2
C3/4, 4/5, 5/6	2

*BMI* Body Mass Index, *ASA* American Society of Anesthesiologists, *VAS* Visual Analog Acale, *JOA*-Japanese Orthopedic Association, *NDI* neck disability index

### Perioperative management

**Fig. 1:**
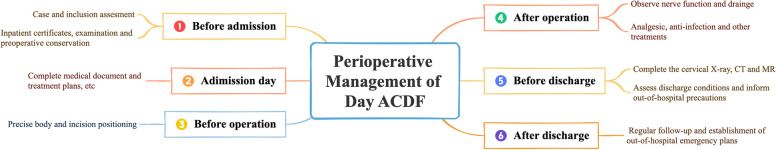
The protocol of the perioperative management of the day ACDF

During the perioperative period, the chief surgeon evaluated the patient’s condition and decided whether day surgery was appropriate in that case. The anesthesia clinic completed the preoperative anesthesia assessment, the rehabilitation doctors informed the relevant rehabilitation matters, and the nurses advised the patient to quit smoking and drinking, performed tracheal push training, and prepared a neck brace. The management details of the Spine Surgery Department are shown in Fig. 1.

### Surgical procedure

General anesthesia was induced in the patients, and neuroelectrophysiological monitoring was conducted. The patients remained in the supine position with the neck slightly extended and received antibiotics half an hour before the incision. Lateral fluoroscopy was used for localization of the appropriate cervical level. An anterior right-side longitudinal incision was made along the medial border of the sternocleidomastoid muscle. Following careful soft tissue dissection, the carotid artery was retracted laterally, and the esophagus and trachea were mobilized medially. Once the prevertebral space was entered, the medial aspect of the longus coli muscles was dissected off the vertebral bodies. Subsequently, anterior decompression was performed, including removal of the ligament, degenerative disc, and osteophytes. A Casper vertebral distracter was placed in the adjacent vertebral bodies to help expose the intervertebral space clearly during the removal of the posterior longitudinal ligament and the osteophytes of the posterior vertebral body. The endplates were abraded by removing the cartilage before fusion, and the bony endplate was preserved as much as possible to prevent implant subsidence. After testing the intervertebral height and width, the appropriate Zero-P allograft implant was implanted in the prepared intervertebral space. After removing the Caspar distracter, self-tapping screws were used cranially and caudally to fix the Zero-P implant. Intraoperative lateral and anterior–posterior fluoroscopic images were obtained, and the position of the implant was adjusted correctly. Hemostasis was ensured, drains were inserted, and the wound was closed with an intradermal suture.

### Postoperative measurement

We used cefuroxime (1.5 g, intravenous infusion, q8h) for 24-h prophylactic anti-infection and flurbiprofen axetil injection (100 mg, intravenous infusion, q12h) for postoperative analgesia. The nursing team was responsible for basic postoperative management of the airway, drainage tube, limb function, and others. The attending doctor or the operator and nurse assessed the patient’s mental condition and limb function 3 h after operation. The wound drainage tube (drainage volume < 5 mL) was removed on the first day after surgery, meanwhile, the cervical spine radiography, CT, and cervical disc MRI were performed. The doctor in charge, nurses and rehabilitation therapists guided patients in postoperative rehabilitation exercises based on the patient’s symptoms and postoperative images. We informed the patients and their families in detail about precautions out-of-hospital when discharged.

### Clinical assessment

The operative time, intraoperative blood loss, postoperative drainage, 30-day hospital readmission rate, and other complications were recorded to evaluate surgical safety. The neurologic status was evaluated using the neck disability index (NDI) and Japanese Orthopedic Association (JOA) score preoperatively and on the discharge day. The JOA recovery rate (RR) was defined according to the rationale by Hirabayashi et al. [19] and calculated as follows: RR = (postoperative JOA score–preoperative JOA score)/(17–preoperative JOA score) × 100. The RR results were grouped as excellent (≥ 75%), good (50%–74%), fair (25%–49%), and poor (< 25%). Neck or arm pain was graded using the 10-point visual analog scale (VAS) at the same time points. The postoperative dysphagia symptoms were assessed by the surgeon depending on the patient’s responses as none, mild, moderate, and severe according to Bazzaz’s standard (Table 3) [20].

**Table 3:** The standard of dysphagia

Symptoms		
Severity	Liquid	Solid
None	None	None
Mild	None	Rare
Moderate	None or rare	Occasionally (only with specific food)
Severe	None or rare	Frequent (majority of solids)

### Radiological evaluation

Anterior–posterior radiography of the cervical spine was conducted to evaluate the cervical curvature using Cobb’s angle. CT was used to evaluate the location of the Zero-Ps implants, and MRI was used to evaluate the neurodecompression on the first day postoperatively. An independent radiologist, who was not a member of the research team, recorded these data and added them to the database.

### Statistical analyses

All statistical analyses were conducted using Statistical Product and Service Solutions, version 27.0 (IBM, USA). The quantitative data were verified for normality using the Shapiro‒Wilk test. Normally distributed quantitative data are presented as means±standard deviations. The quantitative data were compared using the paired-sample t-test. The non-normally distributed are presented as medians and interquartile ranges. These data were compared using the Wilcoxon rank sum test. Categorical data were compared using the chi-square test. *P*-values <0.05 were considered statistically significant.

## Results

### General surgical data

The surgery was successful in all patients, and no patient was lost to follow-up. The follow-up period was at least 1 month. According to the statistical results, the average operation time was 71.1 ± 20.2 min, intraoperative blood loss was 19.1 ± 6.2 mL, and postoperative drainage was 9.6 ± 5.8 mL.

### Clinical outcome

All patients experienced reduced neck or arm pain and neurological recovery postoperatively. Moreover, the postoperative VAS, NDI, and JOA scores improved significantly (*P* < 0.01). The RR was 93.9% in good or excellent, with excellent RR in 11 patients, good RR in 20 patients, with fair RR in 2 patients. The clinical outcomes are shown in Table 4.

**Table 4:** The comparison of VAS, NDI, and JOA score

	Pre-OP	Pos-OP	*P*
VAS	7.1 ± 1.2	3.1 ± 1.3	< 0.001
NDI (%)	66.7 ± 4.8	24.1 ± 2.5	< 0.001
JOA	7.7 ± 1.3	14.2 ± 1.4	< 0.001

V*AS* Visual Analog Scale, *JOA* Japanese Orthopedic Association, *NDI* neck disability index, *OP* Operation

### Radiological findings

**Fig. 2:**
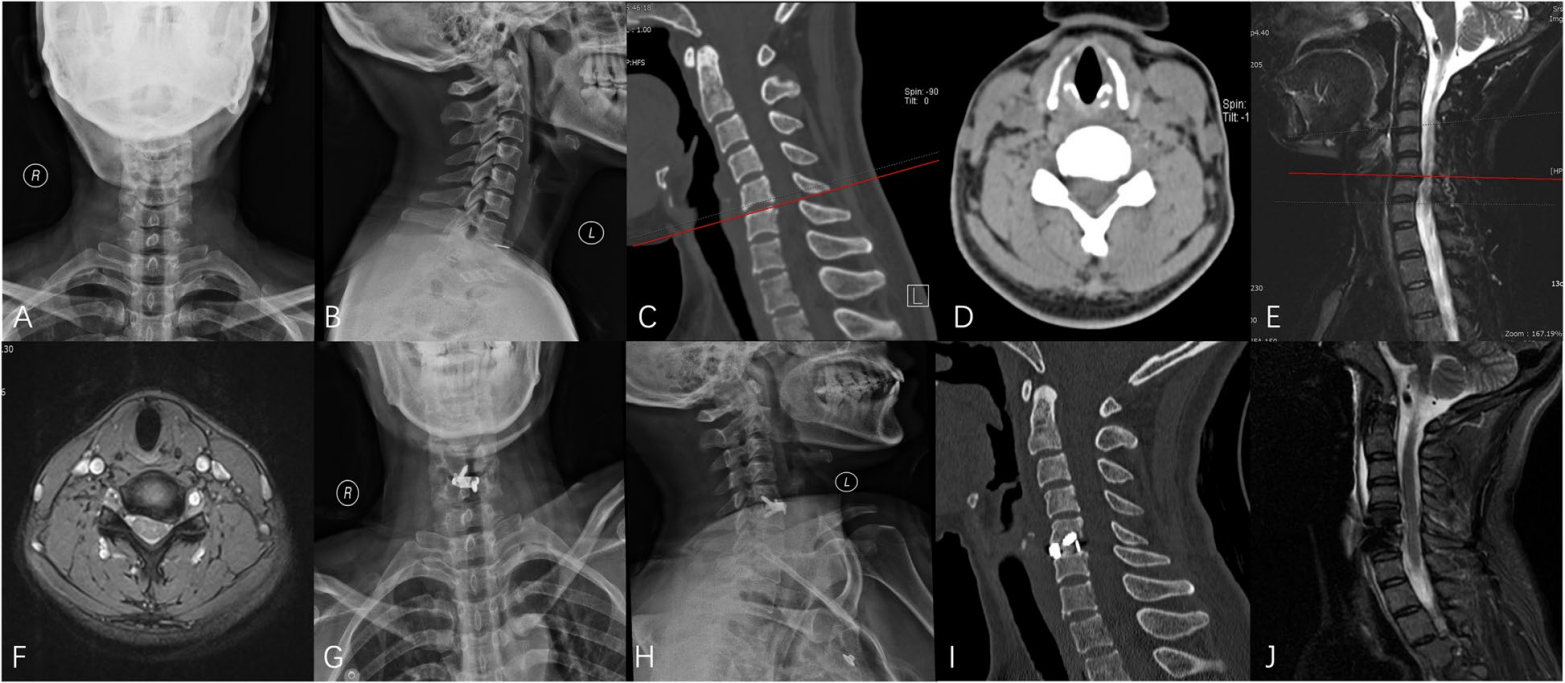
**A**, **B** The preoperative A-P X-Ray showed the height of C5/6 decreased, **C** The preoperative CT sagittal showed cervical lordosis, the height of C5/6 decreased with posterior edge osteophyte hyperplasia, **D** The preoperative CT coronal view showed disc herniation of C5/6, **E**, **F** The preoperative MRI showed disc herniation of C5/6 (left), **G**, **H** The postoperative A-P X-ray showed a good cervical physiological curvature, **I** The postoperative CT showed a perfect position of the Zero-P implant, **J** The postoperative MRI showed the spinal cord obtained a full decompression with smooth cerebrospinal fluid circulation

All implantations were successful, and a perfect cervical curvature was obtained with Cobb’s angle increasing from 14.5° ± 4.0°preoperatively to 26.3° ± 5.4° postoperatively (*P* < 0.01). According to the CT findings, the location of the implants was excellent, with no instrument contacting the anterior, posterior, and bilateral edges of the vertebral body. However, four Zero-Ps were not located in the perfect center in the coronal position. All patients showed sufficient decompression on MRI with complete cervical discectomy and good cerebrospinal fluid circulation. A representative case is shown in Fig. 2.

### Complications

All patients underwent successful surgery with no intraoperative complications. Although no patient was readmitted within 30 days after discharge, one patient experienced an incisional hematoma on the 6th day after discharge, which was cured by pressure dressing. Dysphagia, a prominent postoperative complication, was observed only in one patient (3.0%). The patient reported mild symptoms on the first day postoperatively, which disappeared after 1 week without special treatment.

## Discussion

Since ACDF was first described in 1958 [6], it has become one of the most commonly performed spinal operations [21]. Additionally, it is considered the gold standard procedure for DCS due to its relatively minimal risk, specifications, and reliability [22]. Currently, routine inpatient ACDF surgery is the norm. Outpatient ACDF surgery has been reported in developed countries such as the European countries and the United States [16, 17, 23], and there are only a few reports on day ACDF surgery in China. To the best of our knowledge, our study is the first to report a series of cases on day ACDF procedure to treat DCS. The aim was to analyze the clinical implications, surgical safety, and early effects to provide a better surgical option for eligible patients.

The operation process of all the patients was smooth and successful. The operative time was 71.1 ± 20.2 min, which was similar to or even lower than that reported in previous studies [24, 25]. Additionally, the inoperative blood loss was 19.1 ± 6.2 mL, which corroborated the findings of some studies [21, 26]. Our study reported significant improvement in pain and spinal nerve function postoperatively, which was similar to or even better than the findings of previous studies [25, 27]. Furthermore, the postoperative imaging data confirmed the effect of spinal nerve decompression objectively and revealed a better cervical physiological curvature, with Cobb’s angle improving from 14.5° ± 4.0° preoperatively to 26.3° ± 5.4 ± postoperatively (*P* < 0.01). Thus, this study strongly demonstrates that the safety and early efficacy of the day ACDF procedure are comparable to those of conventional inpatient surgery. Moreover, the hospitalization duration for day ACDF in our research did not exceed 48 h, which significantly shortened the hospitalization time and hospitalization expenses compared to conventional inpatient surgery and greatly reduced the medical financial burden on the patients and health insurance; this was consistent with the advantages of outpatient and some inpatient surgeries reported previously [16, 18]. However, the length of hospital stay did not have an advantage over the outpatient surgeries implemented in developed countries such as the United States and European countries [17, 23]. Hence, future studies are warranted to continue to learn and improve on this aspect.

Although the efficacy and safety of day ACDF surgery are high, the prevention and management of postoperative complications are extremely important because the postoperative observation time in the hospital is not long. Dysphagia, dyspnea, and intraincisional hemorrhage are common short-term complications after ACDF, especially dyspnea and intraincisional hemorrhage that lead to hematoma formation, which may be fatal in severe cases if not treated in a standardized and timely manner. No cases of postoperative dyspnea were reported in our study; one patient had mild postoperative dysphagia, which gradually disappeared without special treatment, and its incidence (3.0%) was significantly lower than that reported previously [20, 28, 29]. Notably, some studies reported that no drainage tubes were placed after ACDF [16]; however, our study concluded that using a trabecular drainage tube could ensure adequate drainage of the operative area to reduce hematoma formation and improve surgical safety. Postoperative drainage should be removed when the drainage is < 5 mL on the first day after the operation or the second postoperative day, as it would not have any adverse effects on the patient. However, one patient presented a delayed incisional hematoma on the 6th postoperative day, though it did not adversely affect breathing or swallowing. The hematoma did not increase in size on the second ultrasound examination performed the next day, and it decreased and disappeared gradually with pressure bandaging and dynamic ultrasound monitoring. After taking a history from the patient and her family, we learned that the patient had moved her neck vigorously without wearing a cervical brace. We hypothesized that the vigorous movement of the neck may have caused the tearing of the initial healing tissue in the incision area, causing delayed bleeding. All the patients in our study were discharged on schedule successfully, with good out-of-hospital recovery and no cases of delayed conversion to regular hospitalization. Furthermore, none of the cases needed to return to the hospital for postoperative treatment. The 30-day readmission rate was significantly lower than that reported previously [13, 23]. Thus, our study concluded that day ACDF has high safety and a low complication rate. The factors described below may have influenced our findings. First, all the patients had a normal body mass index (23.8 ± 2.7 kg/m^2^) with a thin anterior cervical fat layer that could be easily separated during the operation. This helped expose the operative field fully without excessive stretching and reduced the stretching of the esophagus and trachea; thus, the postoperative edema and risk of postoperative dysphagia and respiratory distress were reduced. Second, most of the surgical segments in this study were between C4 and C7, which have the best surgical field of view and reveal the surgical field clearly, allowing surgery completion without excessive stretching. Third, no obvious bone formation was found on preoperative imaging in any patient, and intraoperative decompression caused less interference with the bony mechanism, leading to less intraoperative and postoperative bleeding and postoperative drainage. Fourth, all the internal fixation consumables belonged to the Zero-P series that has the advantage of smooth implantation into the intervertebral space with less soft tissue stripping compared with the traditional titanium plate combined with intervertebral fusion internal fixation system, which is bound to reduce intraoperative bleeding caused by tissue stripping. Meanwhile, the anterior edge of the Zero-P is flush with the anterior edge of the vertebral body, which greatly reduces the irritation to the esophagus and the postoperative dysphagia symptoms. Fifth, the procedure to reduce intraoperative bleeding caused by tissue stripping was performed routinely by the surgical team. Finally, inpatient ACDF has been performed routinely for many years by our department, with rich clinical experience, standardized operation protocols, and short operation time. This can help shorten the intraoperative tissue stretching time and lower the postoperative inflammatory edema, further reducing the occurrence of postoperative dysphagia and other complications.

Some postoperative complications after ACDF, such as anterior cervical hematoma and intralesional hematoma, have a very low incidence, but the consequences can be serious [13]. As the first series of case studies of day ACDF conducted by our team in China, we have formulated strict discharge standards as follows: The attending doctor or operator conducted a detailed physical examination of the patient on the second morning after the operation, including vital signs, condition of the surgical incision, and motor and sensory functions of the lower limbs. The patients were discharged if their anterior neck tension was not high and there was no incision swelling, dyspnea, obvious abnormality in the motor and sensory functions of the limbs, or discomfort such as abnormal neck pain. Additionally, the rehabilitation specialist explained the postoperative rehabilitation exercises in detail. If the patients did not fulfill the aforementioned criteria, the regular hospitalization protocol was followed, and they would be treated actively. Meanwhile, we made some out-of-hospital management measures as follows: We instructed the patients to visit the outpatient spine clinic for follow-up at 1, 3, 6, and 12 months postoperatively. The specialist nurses completed the first remote follow-up 3 days after the operation, mainly to understand the improvement in the patient's preoperative symptoms, assess the postoperative pain and wound conditions, and investigate any early postoperative complications. The nurses informed the operator or attending doctor of the follow-up results. Rehabilitation therapists guided the patients in out-of-hospital rehabilitation. Additionally, we made some plans as described below for serious postoperative complications. We completed at least two more follow-ups within 1 week after the operation and insisted on dynamic and continuous out-of-hospital observation. Next, the patients were given the contact number of our Spine Surgery Department and the doctor in charge to ensure professional medical guidance as soon as possible in case of postoperative complications at home. Finally, we established an emergency medical team comprising a spine surgeon, nurse, and emergency doctor to ensure that patients received timely medical evaluation and professional treatment in case of complications, such as postoperative hematoma and wound infection.

This is our first study to incorporate conventional ACDF into the ambulatory mode, and our preliminary study found that the surgical efficacy and safety were good. The researchers synthesized the clinical experience and summarized the considerations related to the perioperative period as described below. Case selection should strictly conform to the inclusion criteria to minimize the risk of perioperative-related complications. It is recommended to start with single-segment cases and gradually progress to double-segment cases based on the actual clinical results. The short duration of day ACDF hospitalization makes it difficult for most patients to accept due to a general heavy emotion about postoperative accidents, and adequate communication and psychological counseling are extremely important. The intraoperative tissue separation should be gentle with careful dissection to avoid injury to the adjacent blood vessels and nerves, thus reducing the strain on the soft tissues in the operative area as much as possible. Intraoperative hemostasis should be strict, and the treatment of bone structures should be minimized to reduce intraoperative bleeding and postoperative drainage. If bone growth is obvious, the treatment must be strict and thorough. The postoperative retention of the drainage tube is recommended, and incision management is considered the responsibility of dedicated personnel. A good follow-up protocol should be established to facilitate early detection and prevention of related complications.

### Study limitations

Although the findings of this research are unique, it has several limitations. First, the sample size of our study was small, and the results lacked strong generalizability and representativeness; hence, further large-scale studies are warranted. Second, the patients in this group were relatively young with good general conditions and an American Society of Anesthesiologists classification of Grade II, without obvious underlying diseases; hence, there was some selective bias. Third, based on the efficacy of conventional inpatient surgery, our research team attempted to transition to performing day ACDF to save medical costs and social resources; however, our results were only derived from descriptive analyses to provide a new option for some eligible patients. Finally, because this study aimed to evaluate the safety and early efficacy of day ACDF, the follow-up period was short.

## Conclusions

Day ACDF has good safety and early clinical efficacy; therefore, it could be a better choice for eligible DCS patients and could be gradually introduced in qualified hospitals.

## Data Availability

The data are available at a reasonable request from the corresponding author.

## Abbreviations

ACDF: Anterior cervical discectomy and fusion
DCS: Degenerative cervical spondylosis
VAS: Visual analog scale
NDI: Neck disability Index
JOA: Japanese orthopedic association
RR: Recovery rate
CSM: Cervical spondylotic myelopathy
CSR: Cervical spondylotic radiculopathy
ERAS: Enhanced recovery after surgery
ASA: American Society of Anesthesiologists
BMI: Body mass index
CT: Computed tomography
MRI: Magnetic resonance imaging
